# Supplementary material for: Social determinants in the delay of starting colorectal cancer treatment
Source: PLoS One. 2026 Jan 8;21(1):e0338727. doi: 10.1371/journal.pone.0338727 (PMC12782427; doi:10.1371/journal.pone.0338727)
Supplement: Attachment — Submitted filename: Reviewr_PONE_D_25_27816-08.04.2025.pdf [file pone.0338727.s001.pdf]

Title

Social determinants in the delay of starting colorectal cancer treatment

Reviewer's Comments

This manuscript explores the influence of individual, socioeconomic, and health system factors on delays in initiating colorectal cancer (CRC) treatment in Brazil, assessing compliance with Law 12.732/2012. Utilizing a robust dataset of 65,582 CRC cases from 2013 to 2019, the authors employed multilevel Poisson regression to identify associations with treatment delays, concluding that significant sociodemographic and systemic disparities affect timely treatment. The manuscript is well written.

Areas of Improvement

Introduction

1. The introduction presents important global cancer statistics, which help contextualize the broader public health relevance of the study. However, given that the focus of the manuscript is specifically on colorectal cancer (CRC), it would strengthen the rationale if the authors included current CRC specific data on incidence and mortality rates, both globally and within Brazil. Furthermore, integrating statistics that explicitly demonstrate how delays in treatment initiation impact CRC outcomes such as stage progression and survival rates would better convey the magnitude and urgency of the problem. This would enhance the reader's understanding of the study's significance and the policy implications of its findings.
2. **Line 69 pg 4** " ***The literature points that delayed treatment is associated with an increase in mortality for all types of cancer***". Authors did a great work trying to emphasize on the impact of delayed in treatment. However, at this stage, the authors have almost provided the conclusion in the introduction. As such, I suggest they remain focused on articulating the problem with CRC treatment in the country. As previously mentioned, authors should consider supporting their claims with figures.
3. While the manuscript presents a compelling analysis of the association between social determinants and delays in CRC treatment initiation, it would benefit from the inclusion of a stronger theoretical framework to contextualize these relationships.

Methods

1. The authors should provide more detailed justification and explain whether any imputation or sensitivity analysis was conducted to address the potential bias caused by missing data and exclusions.
2. The absence of significant contextual effects in the multilevel models, despite some bivariate associations, warrants further discussion. Are there measurement or aggregation issues? Could variables such as the Gini index or service density mask within-state heterogeneity?
3. Although Poisson with robust SE handles variance inflation, authors can enhance their work be clearly stating how overdispersion was considered and managed.
4. Also, it would strengthen the paper if the authors compared results with standard logistic regression models to demonstrate robustness.

Results

1. The U.S. Preventive Services Task Force (USPSTF) recommends routine colorectal cancer (CRC) screening for adults aged 45 to 75 years. Consequently, CRC cases diagnosed in individuals younger than 45 years fall under the category of early-onset colorectal cancer (EO-CRC). In the current study, the authors grouped patients aged 18–49 years together. However, it would be valuable to further disaggregate this group, particularly to identify and report on individuals aged 18–44 who fall below the USPSTF recommended screening threshold. This information could provide important insights into the burden of EO-CRC within the study population. Given the substantial number of cases in this age range, the authors should consider discussing the implications of EO-CRC for screening policies and how it may contribute to delayed diagnosis and treatment initiation in this younger demographic.
2. The tables are well presented but need titles with the total study sample (N=65,582).
3. Please include a scale bar and a north arrow on your map. These elements enhance its readability and professional look. Also, can you include Caption in Figure 2?

Discussion

1. While the discussion mentions structural and systemic causes for disparities, it would benefit from deeper exploration of the mechanisms behind these factors. For example, how does fragmentation in the referral pathway concretely lead to treatment delays? Are there examples of bottlenecks or specific steps in the process where delays are most acute?

Minor comments

1. P.2, ln 41: Please spell out "42.1%" as "Approximately forty-two percent (42.1%) …"
2. You should revise the title for Table 1.
3. Clearly provide titles for all tables and figures.
4. P.44, ln 407: "Referencias" should be "References"
