# Supplementary material for: Social determinants in the delay of starting colorectal cancer treatment
Source: PLoS One. 2026 Jan 8;21(1):e0338727. doi: 10.1371/journal.pone.0338727 (PMC12782427; doi:10.1371/journal.pone.0338727)
Supplement: Attachment — Submitted filename: Response to Reviewers.pdf [file pone.0338727.s002.pdf]

RESPONSE TO REVIEWERS

INTRODUCTION

1.The introduction presents important global cancer statistics, which help contextualize the broader public health relevance of the study. However, given that the focus of the manuscript is specifically on colorectal cancer (CRC), it would strengthen the rationale if the authors included current CRC specific data on incidence and mortality rates, both globally and within Brazil. Furthermore, integrating statistics that explicitly demonstrate how delays in treatment initiation impact CRC outcomes such as stage progression and survival rates would better convey the magnitude and urgency of the problem. This would enhance the reader's understanding of the study's significance and the policy implications of its findings.

Dear reviewer, thank you for your valuable contribution. We have accepted your suggestion, as demonstrated below. The corresponding changes have been incorporated into the manuscript and can be found between lines 56 and 65.

- Cancer remains a major global health challenge, with approximately 20 million new cases and 10 million deaths reported in 2022. Demographic projections indicate that the annual incidence is expected to reach 35 million cases by 2050, representing a 77% increase. Among specific cancer types, colorectal cancer (CRC) was the third most incident 9.6 cases, and the second leading cause of cancer-related mortality 9.3[1]. In Brazil, the National Cancer Institute (INCA) estimates an incidence rate of 11.43 cases per 100,000 inhabitants for the 2023-2025 period, ranking CRC as the fourth most frequent cancer, while the mortality rate registered in 2021 was 9.6, placing it third[2, 3].
- . In this context, CRC represents a significant health issue, with survival directly related to the stage at diagnosis and timely access to treatment[4].

Studies show that diagnostic delays longer than 30 days are associated with worse five-year overall survival[5], and that each four-week delay in starting treatment increases the risk of mortality by 13% to 15%, potentially reaching 39% with a twelve-week delay[6].

2.Line 69 pg 4 " *The literature points that delayed treatment is associated with an increase in mortality for all types of cancer*". Authors did a great work trying to emphasize on the impact of delayed in treatment. However, at this stage, the authors have almost provided the conclusion in the introduction. As such, I suggest they remain focused on articulating the problem with CRC treatment in the country. As previously mentioned, authors should consider supporting their claims with figures.

Dear reviewer, considering your observation, we decided to remove the paragraph in question and include information that further elaborates on the barriers to timely access to cancer treatment, as demonstrated below. Additionally, the numerical data you requested were incorporated into the paragraph, as indicated in Response 1. These changes can be found between lines 74 and 80 of the manuscript.

- This impact becomes evident in the context of cancer treatment, where structural and social barriers hinder effective access to care. The shortage of infrastructure, specialists, and essential medications, combined with lack of reimbursement and high costs, reflects economic and political inequalities that compromise service delivery[9] Additionally, cultural factors such as fear of treatment side effects and preference for traditional practices, along with low health literacy and misinformation, contribute to treatment non-adherence especially in low- and middle-income countries like Brazil[10-11].

3.While the manuscript presents a compelling analysis of the association between social determinants and delays in CRC treatment initiation, it would benefit from the inclusion of a stronger theoretical framework to contextualize these relationships.

Dear reviewer, thank you for your comment. We would like to inform you that we adopted the theoretical conceptual model of the Social Determinants of Health (SDH), developed by the WHO Commission on Social Determinants of Health, as the basis for structuring the study variables. To enhance clarity, we included an illustrative figure (Figure 1), presented in the Methods section, which represents the organization of the variables according to the model's three dimensions: sociopolitical context, socioeconomic position, and intermediate health determinants. Additionally, we provided an explanation of how this model was applied in the study. These modifications can be found between lines 130 and 142 of the manuscript.

- The independent variables (Figure 1) were organized according to the theoretical model of the Social Determinants of Health (SDH) proposed by the Commission on Social Determinants of Health (CSDH) of the World Health Organization (WHO). This conceptual framework includes three main categories of SDH: sociopolitical context, socioeconomic position, and intermediate health determinants[21].
- In the sociopolitical dimension, variables reflecting the socioeconomic conditions of Brazilian states were considered, such as the Gini Index and the Social Vulnerability Index (SVI). In the structural determinants, individual-level variables representing the socioeconomic position of patients with colorectal cancer (CRC) were included, such as educational

level. Finally, the intermediate determinants comprised biological and behavioral factors, along with local indicators related to the availability of and access to health services, such as age group and oncology service density. Figure 1 illustrates the distribution of these variables according to the aforementioned theoretical model.

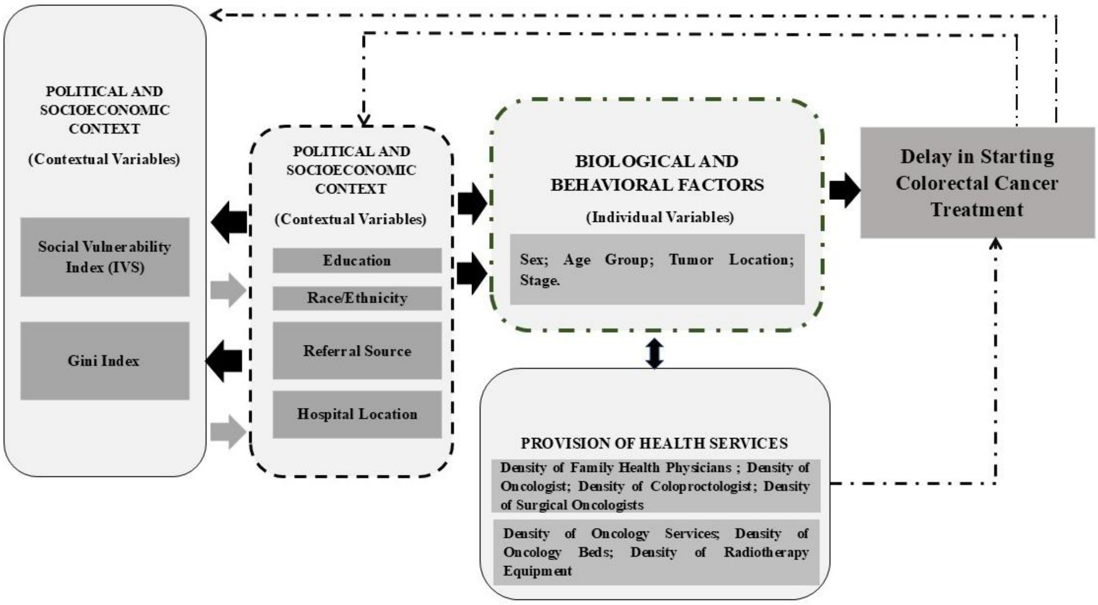

Finally, this theoretical framework was also incorporated into the discussion section to contextualize and interpret the study's findings based on the dimensions of the Social Determinants of Health, as demonstrated below. We believe these changes have strengthened the theoretical foundation of the study and contributed to greater methodological clarity and depth in the interpretation of the results. The corresponding modifications can be found between lines 293 and 300 of the manuscript.

- The delay in initiating CRC treatment, when compared across Brazilian federal units in this study and based on the theoretical model of the Social Determinants of Health (SDH), was associated, at the level of intermediate

determinants, with younger age groups as well as with tumor location in the rectum (biological factor). Regarding socioeconomic position, associations were observed with low educational level, race, location of the treatment hospital, and referral source. No significant associations were found in the sociopolitical context or in the intermediate context related to access to health services.

METHODS

1. The authors should provide more detailed justification and explain whether any imputation or sensitivity analysis was conducted to address the potential bias caused by missing data and exclusions.

We appreciate the reviewer's insightful comment. In our analysis, we adopted a complete-case approach, excluding records with missing data. We recognize that this strategy may introduce bias, and have now included a more detailed justification for this methodological choice in the revised manuscript. Although we did not perform formal imputation or sensitivity analyses, we explicitly acknowledge this limitation and discuss its potential impact on our results.

The missing data are primarily due to incomplete or absent information in medical records, which are the source of the secondary data used. This limitation may affect the completeness and overall quality of some variables analyzed. We have added this consideration to the discussion section and emphasized the need for improved data recording practices in future research and health information systems.

Additionally, for this same database, a previous multilevel analysis assessing data completeness demonstrated that missingness was not random, but rather associated with contextual and individual-level factors. This reinforces the importance of accounting for potential systematic bias in the interpretation of our results and highlights the relevance of targeted actions to improve data quality, which was added to the limitations section of the article.

- This study presents some important limitations that should be considered when interpreting the results. The first relates to the cross-sectional design adopted, which prevents the establishment of temporal relationships between the outcomes investigated and the factors analyzed. In addition, the use of secondary data from Brazil's health information systems represents a methodological limitation, particularly regarding the completeness and quality of the information. Although the RHC stands out as the most comprehensive and systematized source available in the country for cancer diagnosis being essential for epidemiological analyses and widely used in scientific research[62] it is recognized that its data may be subject to errors resulting from manual or incomplete extraction from medical records. Although no formal imputations or sensitivity analyses were performed, this may represent a limitation with potential impact on the findings. Most of the missing data are attributed to gaps in clinical records, which may compromise the quality of some of the variables analyzed.

  For this same database, a previous multilevel analysis assessing data completeness demonstrated that missingness was not random but was associated with contextual and individual-level factors. This reinforces the importance of considering potential systematic bias when interpreting our results and highlights the relevance of targeted actions to improve data quality[52].

2. The absence of significant contextual effects in the multilevel models, despite some bivariate associations, warrants further discussion. Are there measurement or aggregation issues? Could variables such as the Gini index or service density mask within-state heterogeneity?

We appreciate the reviewer's thoughtful observation. The absence of significant contextual effects in the multilevel models, despite some associations observed in the bivariate analyses, may be partially explained by measurement and aggregation limitations. Contextual variables such as the Gini index and service density were included at the state level, which may not adequately capture intra-state variability, especially in large and heterogeneous states. This level of aggregation can indeed mask within-state heterogeneity and attenuate potential contextual effects.

Moreover, some contextual indicators may suffer from limited sensitivity or specificity in capturing the dimensions most relevant to the outcome studied. We have now addressed this limitation more explicitly in the discussion section, acknowledging the potential impact of both measurement error and ecological fallacy on our findings.

- Additionally, the absence of significant contextual effects in the multilevel models, despite some associations observed in the bivariate analyses, may be related to measurement and aggregation limitations of the contextual variables used. Indicators such as the Gini Index and service density were operationalized at the state level, which may not adequately capture intrastate variability, especially in states with large territorial extension and high socio-spatial heterogeneity. This level of aggregation may obscure internal inequalities and attenuate potentially relevant contextual effects. Furthermore, some indicators may have limited sensitivity or specificity to capture key dimensions related to the outcome studied, which introduces risks of measurement error and ecological fallacy.

3. Although Poisson with robust SE handles variance inflation, authors can enhance their work be clearly stating how overdispersion was considered and managed.

We appreciate the reviewer's comment. To address potential overdispersion in the data, we employed a multilevel Poisson model with robust standard errors, which is a well-established approach to correct for variance inflation in count models.

Additionally, we assessed overdispersion by examining model residuals and performing specific tests. The overdispersion test was conducted using the goodness-of-fit statistic based on Pearson's chi-square. For the analysis of individuals who arrived with a diagnosis but without treatment, the obtained value was 23,871.48 with 41,828 degrees of freedom, resulting in a dispersion index (ratio of Pearson chi-square to degrees of freedom) of approximately 0.57. For those who arrived without diagnosis and without treatment, the model presented a log likelihood of -23,417.09 and a significant likelihood ratio chi-square (LR chi2 = 33.15; p < 0.001). The dispersion index (Pearson chi-square divided by degrees of freedom) was about 0.16, indicating absence of overdispersion.

If overdispersion had been detected, we would have considered alternative models such as the multilevel negative binomial model. However, the results obtained with the robust Poisson model were consistent and appropriate for our data, ensuring the reliability of the estimates.

The information about the tests performed, along with their respective results, was added to the study's methodology section.

- Additionally, overdispersion was assessed by examining model residuals and performing specific tests. The overdispersion test was conducted using the goodness-of-fit statistic based on Pearson's chi-square. For the analysis of individuals who arrived with a diagnosis but without treatment, the obtained value was 23,871.48 with 41,828 degrees of freedom, resulting in a dispersion index of approximately 0.57. For those who arrived without diagnosis and without treatment, the test value was 6,892.39 with 41,828 degrees of freedom, resulting in a dispersion index of about 0.16. Since

the dispersion indices for both tests were below 1, this indicates an absence of overdispersion in the analyzed data.

4. Also, it would strengthen the paper if the authors compared results with standard logistic regression models to demonstrate robustness

We appreciate the reviewer's suggestion. However, we would like to highlight that logistic regression models tend to overestimate prevalence ratios when the outcome is common, which can lead to biased interpretations. For this reason, we opted for the Poisson regression model with robust variance, which allows for more accurate direct estimation of prevalence ratios in settings with high outcome prevalence.

Furthermore, for this type of database and considering the methodology employed, our research group prefers to use the Poisson regression model with robust variance, as it is more suitable for the study objectives and the data profile. This approach is widely recognized and well established in the epidemiological literature, having been successfully applied in various studies on multiple types of cancer, both by our group and by researchers worldwide, with publications in prestigious scientific journals. Therefore, the choice of this model aligns with best practices and ensures greater reliability of the estimates obtained.

RESULTS

1. The U.S. Preventive Services Task Force (USPSTF) recommends routine colorectal cancer (CRC) screening for adults aged 45 to 75 years. Consequently, CRC cases diagnosed in individuals younger than 45 years fall under the category of early-onset colorectal cancer (EO-CRC). In the current study, the authors grouped patients aged 18–49 years together. However, it would be valuable to further disaggregate this group, particularly to identify and report on individuals aged 18–44 who fall below the USPSTF recommended screening threshold. This information could provide important insights into the burden of EO-CRC within the study population. Given the substantial number of cases in this age range, the authors should consider discussing the implications of EO-CRC for screening policies and

how it may contribute to delayed diagnosis and treatment initiation in this younger demographic.

We appreciate the reviewer's insightful comment regarding the potential value of disaggregating the 18–49-year-old age group to further highlight individuals aged 18 to 44, who fall below the current USPSTF-recommended threshold for CRC screening.

*We chose to retain the 18–49 age range as a single analytical group, as this is consistent with current international literature and classification, which defines early-onset colorectal cancer (EO-CRC) as CRC diagnosed in individuals younger than 50 years (*Wu CW *et al., 2020; Siegel et al., 2023; NCCN, 2024; USPSTF, 2021;* Cavestro GM*, 2023). Importantly, even cases diagnosed between ages 45 and 49 now within the screening range recommended for average-risk adults are still classified as EO-CRC in recent epidemiological and clinical guidelines.*

Thus, the age grouping used in this study remains aligned with established definitions and does not impact the core findings or the analytical integrity of the results. The focus of our analysis was to understand the social and clinical determinants associated with treatment delays across the EO-CRC spectrum.

Nonetheless, we acknowledge the importance of further emphasizing the subgroup aged under 45, particularly given their exclusion from current routine screening policies. We will therefore revise the discussion section of the manuscript to explicitly address this issue, underscoring the share of cases in this younger group and the implications for delayed diagnosis and treatment initiation.

Furthermore, based on our findings, we will highlight the urgent need to develop a national screening policy in Brazil specifically for EO-CRC, which currently does not exist. Given the global trend of rising EO-CRC incidence and the subsequent revision of international screening recommendations (e.g., lowering the starting age to 45), we argue for a similar reassessment within the Brazilian context especially for vulnerable populations. These insights will be incorporated into the discussion to inform future public health strategies.

Wu CW, Lui RN. Early-onset colorectal cancer: current insights and future directions. World J Gastrointest Oncol. 2022;14(1):230-241. doi:10.4251/wjgo.v14.i1.230.

Siegel RL, Wagle NS, Cercek A, Smith RA, Jemal A. Colorectal cancer statistics, 2023. CA Cancer J Clin. 2023;73(3):233-254. doi:10.3322/caac.21772.

US Preventive Services Task Force, Davidson KW, Barry MJ, et al. Screening for colorectal cancer: US Preventive Services Task Force recommendation statement. JAMA. 2021;325(19):1965-1977. doi:10.1001/jama.2021.6238.

Cavestro GM, Mannucci A, Balaguer F, et al. Delphi Initiative for Early-Onset Colorectal Cancer (DIRECt) International Management Guidelines. Clin Gastroenterol Hepatol. 2023;21(3):581-603.e33. doi:10.1016/j.cgh.2022.12.006

National Comprehensive Cancer Network (NCCN). NCCN Guidelines for Colorectal Cancer Screening. Version 2.2024. Available from: https://jnccn.org/view/journals/jnccn/22/7/article-p438.xml.

This paragraph was added to the discussion to address the requested points. The corresponding modifications can be found between lines 308 and 318 of the manuscript.

- Recent epidemiological studies have consistently shown a concerning increase in the incidence of early-onset colorectal cancer (EO-CRC), defined as cancer diagnosed in individuals younger than 50 years [30–31]. This upward trend is observed globally and here in Brazil, as demonstrated in this study, and represents a significant public health challenge, since EO-CRC patients often present with advanced-stage disease and face delays in diagnosis. In response, organizations such as the USPSTF and NCCN have revised their guidelines, lowering the recommended starting age for screening from 50 to 45 years [32–33]. These changes highlight the importance of including younger populations in screening programs an initiative that should be incorporated in Brazil when a specific program for this condition is implemented within the SUS, aiming to align public health policies with the current scenario.

2.The tables are well presented but need titles with the total study sample (N=65,582).

Dear Reviewer, thank you for your comment. We would like to inform you that the data presented in the tables have been adjusted to reflect the total number of participants in

the study. Table 1 presents a descriptive analysis including the total number of participants, showing the proportions according to the time to treatment initiation (within 60 days and after 60 days). This table reflects the complete dataset (N=65,582) after applying the exclusion criteria. In contrast, Tables 2 (n = 41,838), 3 (n = 23,744), 4 (n = 41,838), and 5 (n = 23,744) refer to analyses based on the stratified dataset. That is, the sample was divided according to whether individuals arrived with or without a diagnosis, while still including all cases of treatment initiation within and after 60 days.

3.Please include a scale bar and a north arrow on your map. These elements enhance its readability and professional look. Also, can you include Caption in Figure 2?

Dear Reviewer *the requested addition has been made as indicated. Regarding the figure captions, we would like to inform you that all captions are included in the body of the manuscript, in accordance with the journal's submission guidelines, placed at the locations where the figures will appear in the final version.*

Specifically:

- *The caption for **Figure 1** can be found between lines **143 and 144**;*
- *For **Figure 2**, between lines **199 and 200**;*
- *And for **Figure 3**, between lines **209 and 210**.*

DISCUSSION

1.While the discussion mentions structural and systemic causes for disparities, it would benefit from deeper exploration of the mechanisms behind these factors. For example, how does fragmentation in the referral pathway concretely lead to treatment delays? Are there examples of bottlenecks or specific steps in the process where delays are most acute?

Dear reviewer, we found your observation pertinent and, in light of it, we expanded the discussion, highlighting the bottlenecks that contribute to delays in the initiation of treatment, contrary to what is established by law. Below, we present the section that was added to the manuscript:

- In this context, the fragmentation of referral flows constitutes a significant barrier to the effectiveness of care networks and directly contributes to delays in treatment initiation, especially in conditions requiring rapid response, such as cancer. Many referral records are still made manually or in disconnected systems, which promotes errors and data loss. Additionally, triage and scheduling suffer from long queues, team overload, and the absence of clear clinical criteria, hindering proper prioritization of cases. Communication between services is weakened by lack of feedback, unanswered messages, and the absence of effective tracking mechanisms [55-56-57]. Logistic problems, such as insufficient transportation, equipment shortages, and geographic barriers, also compromise continuity of care. Added to this is a culture of disorganized handoffs, generating multiple unnecessary referrals. Furthermore, the system's low responsiveness results in "invisible" waiting lists composed of patients who, even after referral, do not receive care, revealing critical failures in coordination and management of care [58-59].

MINOR COMMENTS

1.P.2, ln 41: Please spell out "42.1%" as "Approximately forty-two percent (42.1%) …"

In accordance with the recommendation, we revised the text to spell out the percentage as follows: 'Approximately forty-two percent (42.1%)

2.You should revise the title for Table 1.

The title of Table 1 has been revised to better reflect its content. The new title is:

- Table 1- Descriptive analysis of time to initiation of colorectal cancer treatment according to individual characteristics and contextual variables. Brazil, by state of residence (n = 65,582).

3.Clearly provide titles for all tables and figures.

We reviewed all tables and figures and provided clear, descriptive titles for each to enhance clarity and readability.

4.P.44, ln 407: "Referencias" should be "References"

It was corrected to "References" in accordance with the journal's language requirements.
