# Supplementary material for: Social determinants in the delay of starting colorectal cancer treatment
Source: PLoS One. 2026 Jan 8;21(1):e0338727. doi: 10.1371/journal.pone.0338727 (PMC12782427; doi:10.1371/journal.pone.0338727)
Supplement: Attachment — Submitted filename: RESPONSE TO REVIEWERS.docx [file pone.0338727.s003.docx]

**RESPONSE TO REVIEWERS**

**INTRODUCTION**

**Line 55: the 77% increase should be 75% increase. This is derived by the following quotient: 35 million/20 million=1.75; therefore, a 75% increase in incidence globally.
Line 56-57: Please correct sentence structure. Should read: "..., colorectal cancer (CRC) was the third most common incident cancer, with 9.6 cases/100,000."
Line 57: Please change to "second leading cause of cancer-related mortality, with 9.3 cases/100,000."**

*Dear reviewer, the suggested changes have been made to the manuscript text. The corresponding changes have been incorporated into the manuscript and can be found between lines 55 and 58.*

- Demographic projections indicate that the annual incidence is expected to reach 35 million cases by 2050, representing a 75% increase. Among specific cancer types, colorectal cancer (CRC) was the third most common incident 9.6/100,000 cases, second leading cause of cancer-related mortality, with 9.3 cases/100,000¹.

**General Comments: The introduction provides essential points to understand the significance of the research. The overarching significance is that time-to-treatment initiation is an important factor significantly affecting CRC outcomes, e.g., survival. The study is focused on the Brazilian population and time-to-treatment initiation is actually supported by national law. The research team proposes to examine the "individual, contextual, socioeconomic, and healthcare system factors" associated with time-to-treatment initiation and whether it meets the legal requirements imposed by national law. Given the importance of healthcare system factors to the design and understanding of this study, it would be beneficial to have a paragraph summary describing in general terms the Brazilian healthcare system, since many readers may not have a good understanding of the system. While reading the article, I was initially confused by some of the results since I did not understand how the study cohort could be made up of CRC patients for whom the diagnosis was not known. It was then surmised that individuals probably were initially seen at community hospitals and then referred to a "Cancer Hospital" for further work up and care, so that members of the study cohort were undiagnosed at entry to the "Cancer Hospital." A brief description of the Brazilian healthcare system in the context of this manuscript's methods may have prevented this confusion.**

*Dear reviewer, following your request, we have added an explanation in the Methods section describing how cancer treatment is organized in Brazil. This addition aims to help readers better understand the structure of the healthcare system and the rationale behind our decision to analyze the groups separately. The corresponding changes have been incorporated into the manuscript and can be found between lines 173 and 206.*

- The cancer care network in Brazil integrates actions from both the public and private sectors, covering all stages from prevention to palliative care. This system encompasses Primary Health Care (PHC), home care, and specialized outpatient and hospital services, supported by technical, normative, logistical, and governance structures that ensure coordination and effectiveness of care ^23^. Primary Health Care, through basic health units and Family Health Strategy teams, serves as the main entry point to the Unified Health System (SUS) and is responsible for screening and referring suspected cases to specialized care ^24^. Specialized care, in turn, is provided by various units such as polyclinics, hospital outpatient clinics, regional reference centers, and autonomous services linked to the private sector. These services rely on specialized professionals who confirm diagnoses and monitor patients’ clinical progress, in addition to performing specific tests essential for both diagnosis and treatment monitoring ^25^.

Cancer treatment is multimodal, encompassing surgical interventions, radiotherapy, and chemotherapy, and is characterized by high costs. It is performed in specialized units organized into different levels of complexity. Notable examples include: (1) High-Complexity Oncology Care Centers (CACONs), which provide treatment for all types of cancer, including hematological ones, and may or may not offer pediatric care; (2) High-Complexity Oncology Care Units (UNACONs), which focus on managing the most prevalent cancer types, with or without radiotherapy, hematology-oncology, and/or pediatric oncology services; and (3) hospital complexes composed of general hospitals that perform oncologic surgeries and provide radiotherapy services, acting as complementary units affiliated with CACONs or UNACONs ^26^.

In this context, it is important to highlight that High-Complexity Oncology Care Centers (CACONs) offer a wide range of services, including specialized medical consultations. This structure allows patients who seek these centers directly, without prior referral from other levels of the healthcare network, to receive diagnostic confirmation and initiate treatment more promptly. Conversely, those who obtain a diagnosis in specialized care units, such as polyclinics or hospital outpatient clinics, may experience longer waiting times before the actual start of treatment ^26^.

For this reason, the analyses between groups were conducted separately, distinguishing individuals without treatment at the time of admission who arrived with a prior diagnosis obtained elsewhere in the healthcare network from those who were also without treatment and entered directly into high-complexity hospitals, where they received both diagnosis and treatment within the same services.

**MÉTODOS**

**Line 92: the present study is technically NOT a cross-sectional study since it does not record outcomes at a specific point in time, but rather includes collected data on a cohort of individuals registered in the RHC over the time period 2013-2019.**

*Dear reviewer, we sincerely thank you for your precise and highly relevant observation. We fully agree that the temporal structure of the RHC database is incompatible with a cross-sectional design. Accordingly, we have revised the manuscript to properly classify the study as a longitudinal observational retrospective cohort. This adjustment more accurately reflects the passive follow-up inherent to the oncology care continuum, enabling the establishment of the temporal relationship between exposures and outcomes.*

*The corresponding changes have been incorporated into the manuscript and can be found between lines 93 and 100.*

- A longitudinal observational retrospective cohort study was conducted using secondary data obtained from the Brazilian Cancer Hospital Registry Integrator (RHC). This system contains standardized information on sociodemographic characteristics, hospital care activities, and tumor clinical features of patients with malignant neoplasms treated in oncology-accredited specialized services. Although the RHC does not actively follow individuals over time, patients are passively monitored throughout their oncology care pathways, and the continuous recording of events enables the establishment of the temporal relationship between exposures and outcomes^14^.

*Additionally, we incorporated a paragraph in the Statistical Analysis subsection to justify the use of prevalence ratios in the context of a retrospective cohort design with a binary operationalized outcome. This clarification ensures methodological coherence with the corrected study design. The corresponding changes have been incorporated into the manuscript and can be found between lines 244 and 250.*

- Although the outcome refers to an event that occurs over time (treatment initiation), it was operationalized as a binary variable (>60 days) rather than as a time-to-event measure. Thus, the proportion of delayed cases at the time of analysis reflects a prevalent condition. In this context, PR were estimated using Poisson Regression with robust variance, which provides unbiased and more interpretable measures of association than odds ratios in studies with high outcome frequency, and avoids the inflation of associations commonly observed with logistic regression^28,29^.

**Line 93: the data from the RHC is very important to the outcomes generated by this study. However, characteristics of the RHC are not discussed. How long has the RHC been in existence? How does the RHC collect cancer data? Is data reported to the RHC by all hospitals in Brazil or just "Cancer Hospitals?" Are there rigorous data completeness and quality standards followed by the RHC as it collects data? For example in North America, central cancer registries are ubiquitous in all US states and Canadian provinces. Data collected by these registries is rigorously evaluated against standards of completeness and quality. Does the secondary, administrative data collected by the RHC also need to meet rigorous standards of data completeness and quality? As a non-Brazilian national, how do I know the secondary data collected by the RHC is complete and of high quality? Given the importance of the RHC data to the outcomes generated herein, some discussion needs to address the completeness and quality of the RHC data. When reviewing Figure 2, one notes of the 80,626 total CRC cases in the RHC database for the relevant time period under study, 112 cases were missing age and sex, or 0.14% of the total CRC cases were missing age and sex. This indicates that RHC data is very complete when it comes to registered age and sex for the cohort. However, also in Figure 2, one notes 5,452 cases were missing dates of diagnosis and date of cancer treatment initiation, or 6.8% of individuals registered in this cohort were missing these vital data. The present study is highly dependent on accurate diagnosis date and treatment initiation date data, and having almost 7% of data missing is alarming and raises concerns about the quality of RHC data. Was the missing data mostly from a couple of Federative Units (UF) or was it randomly missing and missing data equally dispersed among UF? In conclusion, given the importance of the publicly-available RHC data to the outcomes generated for this study, there needs to be discussion about how RHC staff ensures the data they collect is complete and of high quality.**

Dear reviewer, in an attempt to meet your request, these pieces of information have been added to the text. *The corresponding changes have been incorporated into the manuscript and can be found between lines 101 and 121.*

- The RHC was established in 1983 by the Brazilian National Cancer Institute (INCA) and was nationally consolidated in 2007 with the creation of the IntegradorRHC system. This registry is responsible for the continuous, systematic, and standardized collection of clinical and epidemiological data on patients with malignant neoplasms treated in public, private, philanthropic, and university hospitals across Brazil. Data collection is conducted using standardized instruments such as the Tumor Registration Form and Follow-up Form, applying internationally recognized coding systems (ICD-10 and ICD-O-3) and the SisRHC software, which includes automated routines for data verification and validation. The periodic submission of information to INCA is mandatory for oncology-accredited hospitals within the Brazilian Unified Health System (SUS), including High-Complexity Oncology Care Centers (CACONs) and High-Complexity Oncology Care Units (UNACONs), as established by Ministerial Ordinances No. 3.535/1998 and No. 741/2005, and voluntary for other institutions^14^.

The RHC system follows rigorous procedures for quality control and data consistency, guided by indicators defined in official INCA manuals, such as variable completeness, internal consistency, and time intervals between diagnosis and treatment. It also implements auditing and validation routines before national database consolidation. These standards and indicators are publicly available on INCA’s official website, ensuring transparency, traceability, and scientific reliability of the data, as well as methodological robustness, standardization, and sound governance for studies that rely on this information^14^.

Regarding the other points raised, in the context of the present study, the high completeness of the main demographic variables (such as age and sex) reflects the robustness of the RHC system. However, approximately 6.8% of the cases lacked information on the dates of diagnosis and treatment initiation. According to INCA’s technical guidelines (*Hospital Cancer Registries – Planning and Management*, 2010; *Hospital Cancer Registries – Routines and Procedures*, 2023), these inconsistencies may result from variations in data entry capacity and infrastructure among oncology centers, rather than systemic deficiencies. Missing data were not concentrated in specific Federative Units (UF) but distributed throughout the country, suggesting random distribution rather than localized data loss. This limitation was acknowledged in the manuscript, and despite these gaps, the RHC remains the most comprehensive and standardized national hospital-based cancer information system in Brazil, supported by continuous validation routines and explicit quality-control mechanisms that ensure the reliability and scientific value of its data.

**Line 97-99: Please change to the following: "The study evaluated cases of malignant neoplasms of the colon (C18), rectosigmoid junction (C19), and rectum (C20), classified according to the 10th revision of the International Classification of Diseases (ICD-10)15."**

*Dear reviewer, the change has been made as requested. The corresponding changes have been incorporated into the manuscript and can be found between line 123.*

- The study included cases of malignant neoplasms of colon cancer (C18), rectosigmoid junction (C19), and rectum (C20), classified according to the 10th revision of the International Classification of Diseases (ICD-10) ^15^. Eligible individuals were aged 18 to 99 years, diagnosed between 2013 and 2019, and monitored in hospital oncology care services in Brazil.

**Line 148-151: This sentence is critical to understanding the analysis plan. Since the study started with diagnosed CRC patients, it does not make sense to divide patients into those with a diagnosis (and no treatment) and those without a diagnosis (and no treatment). This is the importance of providing a brief synopsis of the Brazilian health system in the Introduction of the manuscript. I think what the authors are trying to convey is the study cohort was divided into those with a diagnosis prior to admission to a "Cancer Hospital" This needs to be clarified.**

*Dear reviewer, following your request, we have added an explanation in the Methods section describing how cancer treatment is organized in Brazil. This addition aims to help readers better understand the structure of the healthcare system and the rationale behind our decision to analyze the groups separately. The corresponding changes have been incorporated into the manuscript and can be found between lines 173 and 206, as previously mentioned.*

**RESULTADOS**

**Line 198: change "Among them," to After exclusions, since the subsequent numbers do not add up to 80,626.**

*Dear reviewer, the change has been made as requested. The corresponding changes have been incorporated into the manuscript and can be found between line 260.*

- Between 2013 and 2019, a total of 80,626 colorectal cancer cases were recorded in individuals aged 18 to 99 years. After exclusions, 37.975 (57.9%) accessed treatment within 60 days, while 27.607 (42.1%) experienced a delay in treatment initiation (>60 days after diagnosis) including all cases of people who arrived with and without a diagnosis (Table 1).

**203-204: remove "Brazil, by state of residence" since the table is not stratified by state of residence.**

*Dear reviewer, we respectfully disagree with this suggestion. The expression “by state of residence” cannot be removed because the database, as part of a multilevel analysis, was structured and organized based on the federative unit (state) of residence of the cases obtained from the IRHC. Therefore, this information must be included, as all analyses were conducted using this specific variable. The corresponding changes have been incorporated into the manuscript and can be found between lines 138 and 139.*

- The RHC data were collected by Federative Unit (UF) of residence of the cases and linked to other databases aggregated by UF. Sociodemographic variables were collected from the Atlas of Human Development in Brazil, such as the Gini Index, available from the United Nations Development Programme (UNDP)^17^, and the Vulnerability Index from the Institute for Applied Economic Research (IPEA), all referring to the year 2010^18^.

**Line 213-214: elaborate on the phrase "who arrived with and without a diagnosis". Arrived from where and to where? Perhaps, arrived from a community hospital or other facility to a "Cancer Hospital?"**

*Dear reviewer, as previously requested, we have added information in the Methodology section clarifying the meaning of “who arrived with and without a diagnosis,” which explains the rationale for using this classification. The corresponding changes have been incorporated into the manuscript and can be found between lines 173 and 206.*

- The cancer care network in Brazil integrates actions from both the public and private sectors, covering all stages from prevention to palliative care. This system encompasses Primary Health Care (PHC), home care, and specialized outpatient and hospital services, supported by technical, normative, logistical, and governance structures that ensure coordination and effectiveness of care ^23^. Primary Health Care, through basic health units and Family Health Strategy teams, serves as the main entry point to the Unified Health System (SUS) and is responsible for screening and referring suspected cases to specialized care ^24^. Specialized care, in turn, is provided by various units such as polyclinics, hospital outpatient clinics, regional reference centers, and autonomous services linked to the private sector. These services rely on specialized professionals who confirm diagnoses and monitor patients’ clinical progress, in addition to performing specific tests essential for both diagnosis and treatment monitoring ^25^.

Cancer treatment is multimodal, encompassing surgical interventions, radiotherapy, and chemotherapy, and is characterized by high costs. It is performed in specialized units organized into different levels of complexity. Notable examples include: (1) High-Complexity Oncology Care Centers (CACONs), which provide treatment for all types of cancer, including hematological ones, and may or may not offer pediatric care; (2) High-Complexity Oncology Care Units (UNACONs), which focus on managing the most prevalent cancer types, with or without radiotherapy, hematology-oncology, and/or pediatric oncology services; and (3) hospital complexes composed of general hospitals that perform oncologic surgeries and provide radiotherapy services, acting as complementary units affiliated with CACONs or UNACONs ^26^.

In this context, it is important to highlight that High-Complexity Oncology Care Centers (CACONs) offer a wide range of services, including specialized medical consultations. This structure allows patients who seek these centers directly, without prior referral from other levels of the healthcare network, to receive diagnostic confirmation and initiate treatment more promptly. Conversely, those who obtain a diagnosis in specialized care units, such as polyclinics or hospital outpatient clinics, may experience longer waiting times before the actual start of treatment ^26^.

For this reason, the analyses between groups were conducted separately, distinguishing individuals without treatment at the time of admission who arrived with a prior diagnosis obtained elsewhere in the healthcare network from those who were also without treatment and entered directly into high-complexity hospitals, where they received both diagnosis and treatment within the same services.

**Line 229: remove Brazil, by state of residence since the table is not stratified by state of residence.**

*Dear reviewer, we respectfully disagree with this suggestion. The expression “by state of residence” cannot be removed because the database, as part of a multilevel analysis, was structured and organized based on the federative unit (state) of residence of the cases obtained from the IRHC. Therefore, this information must be included, as all analyses were conducted using this specific variable. As mentioned in a previous comment, this point has already been clarified and further detailed in the Methodology section.*

**In Table 2, under "Density of Oncology Beds (per 100,000 inhabitants), the confidence interval around the category "4.41-10.81" is incorrect. It currently reads "0.77-0.71" whereas the point estimate of 0.79. The point estimate should be within the 95% confidence interval.**

*Dear reviewer, we have made the correction to the appropriate value and thank you for your observation and collaboration.*

**In Table 3, the "Non white" category has an incorrect 95% confidence interval; the confidence interval for the "0.289-0.297" category of the Social Vulnerability Index is incorrect.**

*Dear reviewer, we have made the correction to the appropriate value and thank you for your observation and collaboration.*

**Line 252: the "RP" should probably be PR, so should read PR=0.86.
Line 254 and 255: the "RP" should be PR.
In Table 4, the column heading "RP" should probably be "PR".**

*Dear reviewer, we have made the correction to the appropriate value and thank you for your observation and collaboration.*

**In Table 5, the covariate "Tumor Location" is considered. Why was it not considered in Table 4 analyses?**

*Dear reviewer, Thank you for your observation. The covariate “Tumor Location” was not included in the multilevel model presented in Table 4 because it lost statistical significance during the modeling process and, therefore, was not retained in the final model. In contrast, it is included in Table 5, as it emerged as one of the factors associated with treatment delay for that specific subgroup.*

**DISCUSSION**

**Line 286-290: Would this not be expected if these individuals were diagnosed outside of a "Cancer Hospital?" In my opinion, this says nothing about access to care, but rather is more a product of inefficiency transferring from a community-based facility to a "Cancer Hospital." In fact, beginning at line 303 below, the authors state: "No significant associations were found in the sociopolitical context related to access to health services." Please be consistent in your interpretation or is there something missing in line 286-290 that either indirectly or directly suggests an issue with access to healthcare?**

*Dear Reviewer, we appreciate your thoughtful comment and fully understand the concern regarding the interpretation of this finding. In response, we have added a new paragraph to the manuscript clarifying that this result should not be interpreted solely as a transfer inefficiency between community-based facilities and cancer hospitals. Rather, it reflects broader systemic issues within the organization of Brazil’s Unified Health System (SUS). This addition aims to make the interpretation more consistent and aligned with the structure and functioning of the oncology care network in Brazil. This new paragraph can be found in lines 353 to 361 of the revised manuscript.*

- This result is particularly relevant because, given the way the Brazilian health system is organized especially under the framework of the Network for Chronic Disease Care and the National Policy for Oncology Care it would be expected that a prior diagnosis would facilitate and expedite the start of treatment. However, the findings reveal the opposite: patients diagnosed in other points of the healthcare network face greater barriers to timely access to therapy, highlighting the fragmentation and lack of coordination among levels of care. This scenario suggests failures in communication and care regulation, undermining the comprehensiveness of care and the effectiveness of oncology care pathways^23-26^.

**Line 444: Again, this study did not employ a cross-sectional epidemiologic design, see previous comment.**

*Dear reviewer, thank you once again for your careful evaluation. In accordance with your previous comment, all references to the study design as cross-sectional have been removed or corrected throughout the manuscript to ensure full methodological consistency. The updated paragraph addressing this issue can be found in Lines 514 and 520 of the revised version and reads as follows:*

- This study presents some important limitations that should be considered when interpreting the results. Although the study design is a longitudinal observational retrospective cohort, the outcome was operationalized as a binary variable (>60 days) rather than as a time-to-event measure. Thus, the delay in treatment initiation was analyzed as a prevalent condition, which limits causal inference. Therefore, the associations observed should be interpreted as statistical associations rather than causal effects

*We greatly appreciate your attention to detail, which has helped us ensure full methodological consistency across the revised version of the manuscript.*

**Line 456-460: These are two very important sentences that address a point I brought up in the Methods section above about RHC data completeness and quality. Apparently, missing data was not distributed at random among the UF. This suggests systematic bias in the use of the RHC data that should probably be addressed by more targeted analyses, such as sensitivity analyses.**

*Dear reviewer, we appreciate your observation regarding the potential impact of systematic bias in the RHC database. We agree that this is an important methodological concern. However, as mentioned in the manuscript, a previous multilevel analysis specifically assessing data completeness in the same database demonstrated that missingness was not random but was associated with both contextual and individual-level factors. Considering this evidence, we chose to acknowledge and discuss this limitation rather than perform additional sensitivity analyses, since the primary objective of the present study was to evaluate determinants of treatment delay rather than data quality itself. Nevertheless, we have reinforced this explanation in the Discussion section to make our methodological reasoning clearer.*

*Regarding the other points raised, in the context of the present study, the high completeness of key demographic variables (such as age and sex) reflects the robustness of the Hospital Cancer Registry (RHC) system. However, approximately 6.8% of the cases lacked information on diagnosis and treatment initiation dates. According to INCA’s technical guidelines (Hospital Cancer Registries – Planning and Management, 2010; Hospital Cancer Registries – Routines and Procedures, 2023), these inconsistencies may result from variations in data entry capacity and infrastructure among oncology centers, rather than systemic deficiencies. Missing data were not concentrated in specific Federative Units (UF) but were distributed throughout the country, suggesting a random rather than localized data loss pattern. This limitation was acknowledged in the manuscript, and despite these gaps, the RHC remains the most comprehensive and standardized national hospital-based cancer information system in Brazil, supported by continuous validation routines and explicit quality-control mechanisms that ensure the reliability and scientific value of its data.*

*Furthermore, the RHC has been widely used in numerous studies published across various international databases, demonstrating its scientific relevance and consolidating it as an essential source of information for health planning and management in the field of neoplasms in Brazil.*

**Concluding Comments:**

**I think this manuscript addresses a very important topic in a unique population. As stated by the authors, Brazil has the largest universal public health system in the world, the Unified Health System, making it a very advantageous system for the study of the effects of treatment delay. The results are also very compelling. The main concern is the completeness and quality of the RHC data. There is no discussion of what policies and procedures are used by RHC staff to ensure the collection of complete and high quality data.**

*Dear reviewer, we sincerely appreciate your positive assessment regarding the relevance and contribution of this study, as well as your valuable comment on data quality assurance within the RHC. To address this point, we have expanded the Discussion section of the manuscript to include information on the institutional policies and standardized procedures established by the Brazilian National Cancer Institute (INCA) to ensure data completeness and quality across oncology centers, reinforcing the information already presented in the Methods section of the manuscript regarding the structure and functioning of the RHC, which was also added following a previous suggestion.*

*As described in INCA’s technical manuals (Hospital Cancer Registries – Planning and Management, 2010; Hospital Cancer Registries – Routines and Procedures, 2023), the RHC follows nationally standardized protocols for case registration, coding, and validation. The adopted measures include continuous training of registry staff, centralized audits, periodic consistency checks, and the mandatory electronic submission of data through the IntegradorRHC system. Each oncology unit must periodically submit validated data to INCA, where automatic error detection routines and quality indicators are applied prior to national database consolidation. These procedures aim to maintain data uniformity, minimize missing information, and ensure the reliability of epidemiological analyses based on RHC data.*

*Accordingly, the following paragraph was added to the Discussion section. This new paragraph can be found in lines 545 to 554 of the revised manuscript.*

- Despite these inherent limitations, this study has several strengths that enhance the reliability and generalizability of its findings. It draws on a large, nationally representative dataset derived from the RHC, which operates under standardized data collection, validation, and auditing protocols coordinated by the INCA. The system’s adherence to international coding standards (ICD-10 and ICD-O-3) and continuous quality-control routines ensures high data consistency and scientific reliability. Furthermore, the RHC’s extensive national coverage and its recognized use in numerous epidemiological and policy-oriented studies reinforce the robustness of the present analyses and their contribution to evidence-based planning and management of cancer care in Brazil^14^.

*These additional details were incorporated into the Discussion section to clarify the methodological rigor and institutional framework that underpin the completeness and quality of the RHC database, further strengthening the justification already provided in the Methods section.*
